# Association of *IFNGR1* and *IFNG* genetic polymorphisms with the risk for pulmonary tuberculosis in the Chinese Tibetan population

**DOI:** 10.18632/oncotarget.21413

**Published:** 2017-09-30

**Authors:** Shumei He, Bo Wang, Xikai Zhu, Zhengshuai Chen, Junyu Chen, Demi Hua, Deji Droma, Wensheng Li, Dongya Yuan, Tianbo Jin

**Affiliations:** ^1^ Key Laboratory for Molecular Genetic Mechanisms and Intervention Research on High Altitude Disease of Tibet Autonomous Region, School of Medicine, Xizang Minzu University, Xianyang, Shaanxi 712082, China; ^2^ Key Laboratory of High Altitude Environment and Genes Related to Diseases of Tibet Autonomous Region, School of Medicine, Xizang Minzu University, Xianyang, Shaanxi 712082, China; ^3^ Key Laboratory for Basic Life Science Research of Tibet Autonomous Region, School of Medicine, Xizang Minzu University, Xianyang, Shaanxi 712082, China; ^4^ Department of The 4th Internal Medicine, Xi'an Chest Hospital, Xi'an TB&Thoracic Tumor Hospital, Xi'an, Shaanxi 710100, China; ^5^ School of Life Sciences, Northwest University, Xi’an, Shaanxi 710069, China; ^6^ The Second Affiliated Hospital of Inner Mongolia Medical University, Hohhot, Inner Mongolia Autonomous Region 010030, China; ^7^ Department of Lung, The Third Hospital of Tibet Autonomous Region, Lhasa, Tibet 850000, China

**Keywords:** pulmonary tuberculosis, single nucleotide polymorphisms, *IFNGR1*, *IFNG*, haplotype

## Abstract

Interferon-gamma (IFNG) and its receptor (IFNGR1) are principal genes that associated with tuberculosis. In the current study we aimed to explore the genetic association of polymorphisms of *IFNG* and *IFNGR1* with the risk of pulmonary tuberculosis (PTB) in the Chinese Tibetan population. We selected 467 PTB patients and 503 healthy controls to genotype 9 single nucleotide polymorphisms (SNPs). The unconditional logistic regression analysis was applied for assessing the associations, and the risk of PTB were evaluated by calculating the odds ratio (OR) and 95% confidence interval (CI). The results showed that mutants of rs9376268, rs1327475 and rs1327474 in *IFNGR1* played a protective role in the PTB risk under genotype, dominant and additive model (*P*<0.05). On the contrary, minor allele “A” of rs2069705 in *IFNG* significantly increased the risk of PTB under genotype, dominant and additive model (*P*<0.05). However, after Bonferroni's multiple adjustment was applied to our data, which level of significant was set at *P*<0.0011 (0.05/45). Only variant of rs9376268 was significantly associated decrease the PTB susceptibility under additive model (OR=0.73, 95%CI=0.61-0.88, *P*<0.001). Furthermore, in the haplotype analysis, we found that the haplotypes “C-G-G-A-C”, “C-G-A-G-T” and “T-A-G-G-T” of rs9376267-rs9376268-rs1327475-rs7749390-rs1327474 block were extremely decreased the PTB risk (*P*<0.01), however, the haplotypes “C-G-G-A-T”, “T-G-G-G-T” and “C-G-G-G-T” of the block were extremely increased the PTB risk (*P*<0.01). These results suggested that variants of *IFNGR1* may have a close relation with the PTB risk in Chinese Tibetan population.

## INTRODUCTION

Globally, tuberculosis (TB) remains to be a vital public health problem as well as leading cause of morbidity and mortality [1, 2]. On the basis of the annual report on global control of TB from WHO, approximately 8.6 million new cases occurred in 2012 [3]. Pulmonary tuberculosis (PTB), a pulmonary infectious diseases caused by *Mycibacterium tuberculosis*, which is the most common location, and the source of the infection is mainly through the respiratory tract.

Epidemiological data showed that only about one-tenth of the population infected by Mycobacterium tuberculosis will develop clinical tuberculosis [4]. Twin study of *Mycibacterium tuberculosis*-infected individuals revealed that latently-infected monozygotic twins are more likely to develop pulmonary TB than latently infected dizygotic twins [5], and there are momentous racial differences in tuberculosis incidence. A Genome wide linkage and association study (GWAS) has found 8 independent loci located within or near the immune signaling genes associate with the PTB susceptibility in Indonesians, containing *JAG1, DYNLRB2, EBF1, TMEFF2, CCL17, HAUS6, PENK* and *TXNDC4* [6]. Moreover, study have indicated that genetic factors play an important role in the pathogenesis of tuberculosis [7].

In the past decades, scenic progress have been made in our understanding of the innate and adaptive immunity in the human host defend to PTB [8], and importantly some single nucleotide polymorphisms (SNPs) in congenital immunity genes have been reported to be candidate biomarkers associated with susceptibility to PTB [9]. Cytokine interferon-γ (IFN-γ), encoded by *IFNG* gene, is type II interferon plays a role in the immune response against viral and intracellular bacterial infections, and has been implicated as a mediator in auto-inflammatory and auto-immune disorders [10]. The receptor for INF-γ is constituted of two integral membrane proteins α and β subunits, and α subunit, encoded by *IFNGR1* gene, regulates the potency of IFN-γ signaling. Study have reported the association between polymorphisms of *IFNGR1* and *IFNG* and the risk of PTB in Chinese Han population, and indicated variants in *IFNGR1* gene involved in the PTB risk [11]. However, to the best of our knowledge, there is no report regarding the possible association between polymorphisms of *IFNGR1* and *IFNG* with the PTB susceptibility of Chinese Tibetan population.

In order to investigate potential relationships between *IFNGR1* and *IFNG* SNPs, genotypes, haplotypes, and their role in the etiology of PTB in a Chinese Tibetan population, we performed a comprehensive association analysis in a case-control study. We genotyped five SNPs in *IFNGR1*: rs9376267, rs9376268, rs1327475, rs7749390 and rs1327474; and four SNPs in *IFNG*: rs2069727, rs1861493, rs1861494 and rs2069705.

## RESULTS

### Primers and sample characteristics

A total of 971 Tibetan participants were recruited in this study, including 467 PTB cases and 504 controls. The primers of the nine selected SNPs were listed in Table 1, which were designed by Sequenom MassARRAY Assay Design 4.0 Software. The characteristic of the controls and cases were listed in Table 2, and after difference analysis, result showed that the distribution of gender and age between case and group were good matched.

**Table 1:** Sequence of oligonucleotide primers used in the study

SNP-ID	2nd-PCRP	1st-PCRP	UEP-SEQ
rs9376267	ACGTTGGATGAGATTGA ACAATGGAGCCAC	ACGTTGGATGTGTAA AAAGCCCTGCACACC	CATTCCAGTTTCATCAACA
rs9376268	ACGTTGGATGCCATCT CTGGTTCTCTAAGC	ACGTTGGATGAAGGCT ACTCTGGCAAGAAC	acCTAAGCAACTTCCTTGTAG
rs1327475	ACGTTGGATGTGTTCAA CTTTTGCAGTGGC	ACGTTGGATGGAAGAC TATTTTCTGGTGAC	gcagtAAACATTACCTGAAGCAGATG
rs7749390	ACGTTGGATGTCTGG GGCCGTCCTCAGGTA	ACGTTGGATGTAGGGC GACCTCGGAGAAG	ctcaGTCCTCAGGTACCGTCG
rs1327474	ACGTTGGATGCTTCTC AGCAATTCAGTGTC	ACGTTGGATGAGAGAGG TAAGAGAGCAGAC	ATTCAGTGTCAAATCAGTTTAT
rs2069727	ACGTTGGATGACTG GTGTTTGCCAGCATTG	ACGTTGGATGATTTCT AGCCCCTTCTCCAC	GGAGAGGAAGATTCTGAAA
rs1861493	ACGTTGGATGTTG GAGCAAAGAAGGTCATC	ACGTTGGATGGTGATGA ATCACATGGCTGG	cctGTCATCAAACTTATACAGTGA
rs1861494	ACGTTGGATGAGGGACA ATGAGAGAACTGC	ACGTTGGATGAGGTGA GTTGACAAATCCAG	TGCTTCTCAGTACTCCC
rs2069705	ACGTTGGATGAGGA GACTGAGTCATAGAAG	ACGTTGGATGGGGCAA ACTTGATTCCTGAC	TGAGTCATAGAAGATTTAAGAAG

**Table 2:** Characteristic of the control individuals and patients with pulmonary tuberculosis

Characteristic	Case (N=467)	Control (N=503)	*P*-value
Gender (%)			0.947^a^
female	287 (61.5%)	308 (61.2%)	
male	180 (38.5%)	195 (38.8%)	
Mean age ± SD	50.67±7.8	50.34±7.74	0.508^b^

*P*^a^ was calculated by Pearson Chi-Square test; *P*^b^ was calculated by Welch's t test. *P*<0.05 indicates significant difference.

### SNP analysis

Nine SNPs in the two genes analyzed in this study were successfully genotyped in all cases and controls. Chromosomal position, gene, miner allele frequency (MAF), and HWE test results for candidate SNPs were presented in Table 3. Exact test was used to calculated whether the SNPs departed from the Hardy–Weinberg equilibrium (HWE), and the results showed that none of the SNPs displayed significant deviation from HWE (*P*>0.05). We used chi-square test to assess the influence of gene polymorphism of PTB risk in the allele model, and found that 3 SNPs in *IFNGR1* significantly reduced PTB risk: rs9376268 (OR=0.73, 95%CI=0.61-0.88, *P*=0.001), rs1327475 (OR=0.62, 95%CI=0.44-0.86, *P*=0.005) and rs1327474 (OR=0.54, 95%CI=0.35-0.84, *P*=0.006). We also discovered that rs2069705 in *IFNG* significantly increased PTB risk (OR=1.36, 95%CI=1.1-1.69, *P*=0.005). Other SNPs had no relation with PTB susceptibility.

**Table 3:** Allele frequencies of candidates SNPs examined in *IFNG* and *IFNGR1* gene among the cases and controls

SNP ID	Position	Band	Gene	Role	Alleles	HWE-*P*^a^	MAF		OR(95% CI)	*P*^b^
					A/B		Case	Control		
rs9376267	137531031	6q23.3	IFNGR1	Intron	T/C	0.715	0.39	0.426	0.86(0.72-1.04)	0.117
rs9376268	137532751	6q23.3	IFNGR1	Intron	A/G	1	0.331	0.403	0.73(0.61-0.88)	0.001^*^
rs1327475	137536455	6q23.3	IFNGR1	Intron	A/G	1	0.063	0.098	0.62(0.44-0.86)	0.005^*^
rs7749390	137540370	6q23.3	IFNGR1	Intron (boundary)	A/G	0.53	0.499	0.456	1.19(0.99-1.42)	0.06
rs1327474	137541075	6q23.3	IFNGR1	Promoter	C/T	0.693	0.033	0.06	0.54(0.35-0.84)	0.006^*^
rs2069727	68548223	12q15	IFNG	Downstream	C/T	0.554	0.154	0.13	1.22(0.94-1.57)	0.131
rs1861493	68551196	12q15	IFNG	Intron	G/A	0.514	0.373	0.397	0.9(0.75-1.09)	0.277
rs1861494	68551409	12q15	IFNG	Intron	C/T	0.576	0.358	0.392	0.86(0.72-1.04)	0.122
rs2069705	68555011	12q15	IFNG	Promoter	A/G	0.676	0.253	0.199	1.36(1.1-1.69)	0.005^*^

SNPs: Single nucleotide polymorphisms; A: Miner alleles, B: Major alleles; MAF: Minor allele frequency; HWE: Hardy-Weinberg equilibrium; OR: Odds ratio; CI: Confidence interval. ^a^*P* values were calculated using exact test; ^b^*P* values were calculated using Chi-square test. ^*^P<0.05 indicates statistical significance. Bonferroni's multiple adjustment was applied to the level of significance, which was set at *P*<0.0011 (0.05/45).

Results of genotype model association analyses used unconditional logistic regression analysis were presented in Table 4 and Table 5. In Table 4, we found that under dominant model, three SNPs exhibited a protective role in PTB risk, containing rs9376268 (OR=0.68, 95%CI=0.53-0.88, *P*=0.004), rs1327475 (OR=0.63, 95%CI=0.44-0.9, *P*=0.01) and rs1327474 (OR=0.53, 95%CI=0.33-0.83, *P*=0.006); but rs2069705 was observed to be associated with increased PTB risk (OR=1.42, 95%CI=1.10-1.83, *P*=0.008). Under recessive model, only rs9376268 was associated with lower PTB risk (OR=0.64, 95%CI=0.44-0.93, *P*=0.019). Under additive model, we noticed that the three SNPs rs9376268 (OR=0.73, 95%CI=0.61-0.88, *P*<0.001), rs1327475 (OR=0.61, 95%CI=0.43-0.86, *P*=0.005), and rs1327474 (OR=0.55, 95%CI=0.35-0.85, *P*=0.007) were associated with decreased PTB risk. Contrarily, rs2069705 (OR=1.37, 95%CI=1.1-1.71, *P*=0.004) be associated with increased PTB risk under additive model.

**Table 4:** Logistic regression analyses of association between nine SNPs and pulmonary tuberculosis risk under dominant, recessive and additive model

SNP-ID	Dominant Model		Recessive Model		Additive Model	
	OR(95%CI)	*P*	OR(95%CI)	*P*	OR(95%CI)	*P*
rs9376267	0.84(0.64-1.1)	0.203	0.79(0.56-1.12)	0.188	0.86(0.72-1.04)	0.113
rs9376268	0.68(0.53-0.88)	0.004^*^	0.64(0.44-0.93)	0.019^*^	0.73(0.61-0.88)	<0.001^*^
rs1327475	0.63(0.44-0.9)	0.01^*^	-	0.999	0.61(0.43-0.86)	0.005^*^
rs7749390	1.33(1-1.78)	0.052	1.2(0.88-1.63)	0.249	1.2(1-1.44)	0.054
rs1327474	0.53(0.33-0.83)	0.006^*^	0.54(0.05-5.95)	0.613	0.55(0.35-0.85)	0.007^*^
rs2069727	1.23(0.92-1.64)	0.157	1.41(0.61-3.25)	0.418	1.21(0.94-1.56)	0.137
rs1861493	0.87(0.67-1.12)	0.276	0.89(0.62-1.28)	0.527	0.9(0.75-1.09)	0.271
rs1861494	0.84(0.65-1.09)	0.191	0.79(0.54-1.15)	0.215	0.86(0.71-1.04)	0.117
rs2069705	1.42(1.1-1.83)	0.008^*^	1.72(0.94-3.15)	0.08	1.37(1.1-1.71)	0.004^*^

SNP: Single nucleotide polymorphisms. ^a^*P* values were calculated by unconditional logistic regression analysis. ^*^P < 0.05 indicates statistical significance. Bonferroni's multiple adjustment was applied to the level of significance, which was set at *P* < 0.0011 (0.05/45).

**Table 5:** Frequency distributions of genotypes and their association with the risk of developing PTB

SNP-ID	Genotype	No. (frequency)		OR(95%CI)	*P*^a^
		Case	Control		
rs9376267	C/C	168(36.44%)	162(32.53%)	1	
	T/C	226(49.02%)	248(49.8%)	0.88(0.66-1.16)	0.368
	T/T	67(14.53%)	88(17.67%)	0.73(0.5-1.08)	0.115
rs9376268	G/G	209(44.75%)	179(35.59%)	1	
	A/G	207(44.33%)	243(48.31%)	0.73(0.56-0.96)	0.023^*^
	A/A	51(10.92%)	81(16.1%)	0.54(0.36-0.81)	0.003^*^
rs1327475	G/G	408(87.37%)	409(81.31%)	1	
	A/G	59(12.63%)	89(17.69%)	0.66(0.47-0.95)	0.025^*^
	A/A	0(0%)	5(0.99%)	-	0.998
rs7749390	G/G	109(23.34%)	145(28.83%)	1	
	A/G	250(53.53%)	257(51.09%)	1.29(0.96-1.75)	0.096
	A/A	108(23.13%)	101(20.08%)	1.42(0.98-2.06)	0.06
rs1327474	T/T	437(93.58%)	445(88.47%)	1	
	C/T	29(6.21%)	56(11.13%)	0.53(0.33-0.84)	0.007^*^
	C/C	1(0.21%)	2(0.4%)	0.51(0.05-5.64)	0.582
rs2069727	T/T	336(71.95%)	382(75.94%)	1	
	C/T	118(25.27%)	111(22.07%)	1.21(0.9-1.63)	0.212
	C/C	13(2.78%)	10(1.99%)	1.48(0.64-3.41)	0.361
rs1861493	A/A	182(38.97%)	179(35.59%)	1	
	G/A	222(47.54%)	249(49.5%)	0.88(0.67-1.15)	0.348
	G/G	63(13.49%)	75(14.91%)	0.83(0.56-1.22)	0.341
rs1861494	T/T	189(40.47%)	183(36.38%)	1	
	C/T	222(47.54%)	246(48.91%)	0.87(0.67-1.15)	0.332
	C/C	56(11.99%)	74(14.71%)	0.73(0.49-1.1)	0.13
rs2069705	G/G	259(55.46%)	321(63.82%)	1	
	A/G	180(38.54%)	164(32.6%)	1.36(1.04-1.78)	0.024^*^
	A/A	28(6%)	18(3.58%)	1.93(1.04-3.56)	0.036^*^

SNPs: Single nucleotide polymorphisms; OR: Odds ratio; CI: Confidence interval. ^a^*P* values were calculated by unconditional logistic regression analysis. ^*^P<0.05 indicates statistical significance. Bonferroni's multiple adjustment was applied to the level of significance, which was set at *P*< 0.0011 (0.05/45).

In Table 5, the rs9376268 mutant genotypes “A/A” and “A/G” compared to the wild genotype “G/G” significantly decreased the PTB risk (“A/A” OR=0.54, 95%CI=0.36-0.81, *P*=0.003; “A/G” OR=0.73, 95%CI=0.56-0.96, *P*=0.023). The significantly protective effects were also found in the rs1327475 variant genotype “A/G” and rs1327474 variant genotype “C/T” (rs1327475 “A/G” *vs* “G/G”, OR=0.66, 95%CI=0.47-0.95, *P*=0.025; rs1327474 “C/T” *vs* “T/T”, OR=0.53, 95%CI=0.33-0.84, *P*=0.007). Additionally, rs2069705 mutant genotype “A/A” and “A/G” compared to the wild genotype “G/G” were associated with a statistically significantly increased the PTB risk (“A/A” OR=1.93, 95%CI=1.04-3.56, *P*=0.036; “A/G” OR=1.36, 95%CI=1.04-1.78, *P*=0.024). However, after Bonferroni's multiple adjustment applied to our data, we only found rs9376268 was significantly associated with susceptibility of PTB under additive model.

### Haplotype association analysis

**Figure 1:**
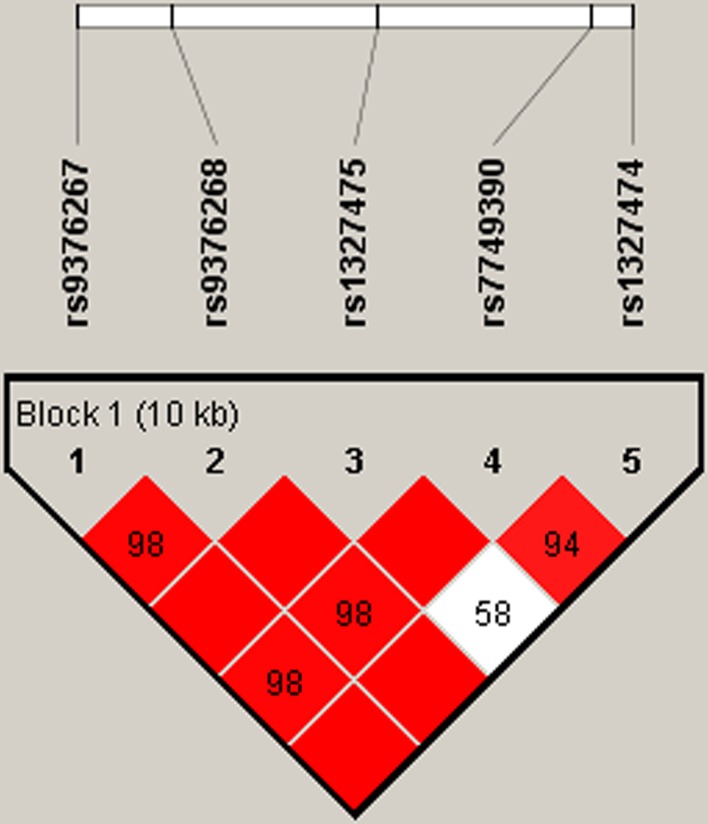
Haplotype block map for part of SNPs in the IFNGR1 gene One block in the figure showed higher LD in the haplotype map, and the block was composed of five SNPs: rs9376267, rs9376268, rs1327475, rs7749390 and rs1327474. The D value was 1, and these five SNPs tended to be co-inherited.

**Figure 2:**
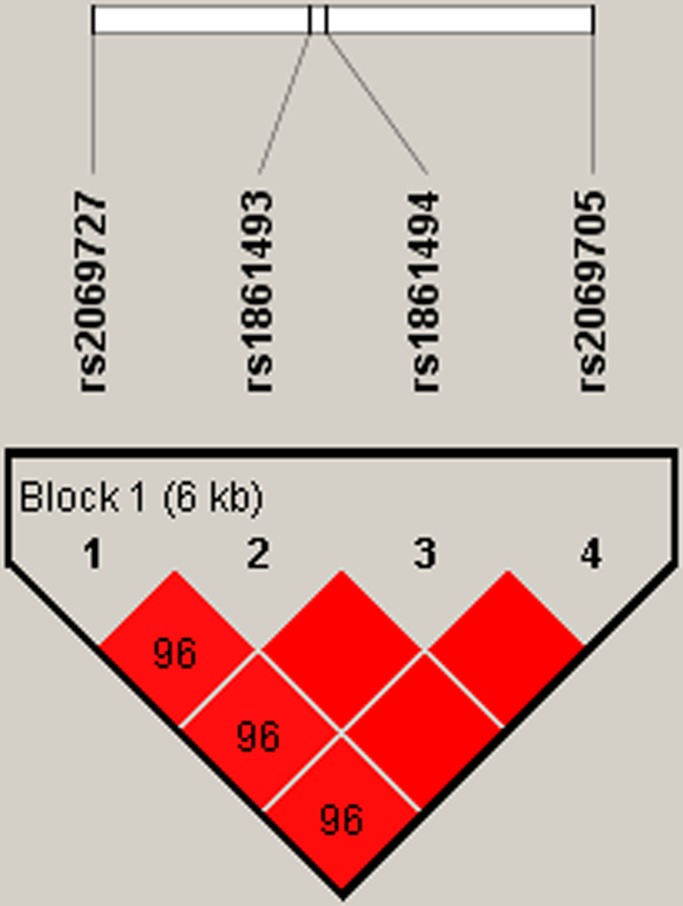
Haplotype block map for part of SNPs in the *IFNG* gene Haplotype-Block Map for *IFNG* based on SNPs rs2069727, rs1861493 rs1861494 and rs2069705 which were included in the block. That the D value was 0.96 indicated strong linkage disequilibrium between the two SNPs.

Using haplotype analysis, two blocks were respectively detected among the *IFNGR1* and *IFNG* SNPs (Figure 1, Figure 2). In block 1 for *IFNGR1* gene, a pair of five SNPs had linkage disequilibrium (LD): rs9376267, rs9376268, rs1327475, rs7749390 and rs1327474), while the block 2 for *IFNG* gene included four closely linked SNPs rs2069727, rs1861493, rs1861494 and rs2069705. The association between haplotype and the PTB risk was evaluated by unconditional logistic regression analysis and the result was showed in Table 6. For the block 1, we discovered three haplotypes associated with an depressed PTB risk: “C-G-G-A-C” (OR=0.52, 95%CI=0.33-0.82, *P*=0.005), “C-G-A-G-T” (OR=0.62, 95%CI=0.44-0.88, *P*=0.007) and “T-A-G-G-T” (OR=0.75, 95%CI=0.62-0.9, *P*=0.002), while another three haplotypes was found associate with an elevated PTB risk: “C-G-G-A-T” (OR=1.36, 95%CI=1.13-1.64, *P*=0.001), “T-G-G-G-T” (OR=2.2, 95%CI=1.38-3.52, *P*<0.001) and “C-G-G-G-T” (OR=2.61, 95%CI=1.5-4.56, *P*<0.001). In the meantime, we just found one haplotype “T-A-T-A” in block 2 had a modest association with increasing PTB risk (OR=1.39, 95%CI=1.01-1.92, *P*=0.043).

**Table 6:** Haplotype frequencies and their associations with PTB risk

Gene	SNPs	Haplotype	Frequency		OR	95% CI	*P*^a^
			Case	Control			
IFNGR1	rs9376267|rs9376268|rs1327475|rs7749390|rs1327474	C-G-G-A-C	0.031	0.059	0.52	0.33-0.82	0.005^*^
		C-G-G-A-T	0.468	0.396	1.36	1.13-1.64	0.001^*^
		C-G-A-G-T	0.064	0.097	0.62	0.44-0.88	0.007^*^
		T-A-G-G-T	0.331	0.398	0.75	0.62-0.9	0.002^*^
		T-G-G-G-T	0.058	0.027	2.2	1.38-3.52	<0.001^*^
		C-G-G-G-T	0.046	0.018	2.61	1.5-4.56	<0.001^*^
IFNG	rs2069727|rs1861493|rs1861494|rs2069705	C-A-T-A	0.154	0.127	1.24	0.96-1.6	0.094
		T-A-T-A	0.097	0.072	1.39	1.01-1.92	0.043^*^
		T-G-C-G	0.357	0.389	0.87	0.72-1.05	0.139
		T-A-T-G	0.376	0.405	0.88	0.73-1.06	0.186

^a^*P* values were calculated by unconditional logistic regression analysis. ^*^P < 0.05 indicates statistical significance.

## DISCUSSION AND CONCLUSION

In the current study, we investigated the potential relationships between 9 SNPs from *IFNGR1* and *IFNG* and risk of PTB in 467 cases and 503 healthy controls subjects of the Chinese Tibetan population. The result of genotype model analysis showed that rs9376268, rs1327475, rs1327474 and rs2069705 had a significantly correlation with the risk of PTB (*P*<0.05). Moreover, rs9376268 still associated with the PTB risk after Bonferroni's multiple adjusted (*P*<0.001). In the haplotype analysis, we found that the haplotypes with the frequency more than 1% in the rs9376267-rs9376268-rs1327475-rs7749390-rs1327474 block were extremely significant associated with the PTB risk (*P*<0.01). These results suggested that variants of *IFNGR1* may have a close relation with the PTB risk in Chinese Tibetan population.

*INFGR1* gene, located in 6q23.3, consists of seven exons and encodes ligand binding chain (alpha) of the interferon-gamma receptor which plays a crucial role in receptor trafficking and signal transduction [12]. Evidence have identified that *IFNGR1* plays an crucial role in regulating immune response against *Mycobacterium tuberculosis* [13, 14]. Moreover, Defects in human *IFNGR1* have been shown to be associated with dominant susceptibility to mycobacterial infection [15]. The ethnic and racial differences in the vulnerability of humans to PTB with respect to *IFNGR1* and *IFNG* polymorphisms have been reported in many races, but not in Chinese Tibetans. For example, Naderi M et al, showed that rs7749390 polymorphism of *IFNGR1* was significantly decreased the PTB risk, but this association was not found in rs1327474 in Zahedan, Southeast Iran [16]. Lü et al. investigated rs1327475 and rs7749390 polymorphisms in PTB patients and found that the rs1327475 polymorphisms increased the PTB risk, while rs7749390 variant reduced the risk PTB in Chinese Han population [11]. Shin et al. performed a correlation analysis between polymorphisms of rs9376268 and rs9376267 and the PTB risk, and revealed that these two loci variants were marginally increased the PTB risk in the Korean population [17].

Here, we examined whether polymorphism of *IFNGR1* were associated with the incidence of PTB in the Chinese Tibetans. From the single locus association analysis, we found that rs9376268, rs1327475 and rs1327474 variants decreased the PTB risk, and the similar association for these three loci under dominant and additive model was also identified. Unfortunately, after Bonferroni's correction was applied to our data, the association was attenuated, and if the sample size is larger, this association may be enhanced. The discrepancy between studies regarding the effect of *IFGNR1* variants on PTB risk may be due to the ethnic and regional differences.

*IFNG* gene, located in 12q15, encodes a soluble cytokine which is secreted by cells of both innate and acquired immune system. Study found that IFN-γ played a critical role in TB protection, and the quantitative parameters of IFN-γ secretion inflected the activity of the TB infectious process [18, 19]. It is reported that study have investigated the variants of *IFNG* for PTB susceptibility in north Indians, and rs1861494 and rs1861493 mutations had been found to be a risk factor for the developing PTB [20]. Variant of rs2069727 was found associated with increase the risk of hepatocellular carcinoma [21], and study had identified the correlation of rs2069705 with lupus erythematosus susceptibility in Chinese Han population [22]. In this present study, we investigated this association in Chinese Tibetans, and our result showed that the mutations of these loci were relatively weak with the PTB risk. Although this study had sufficient statistical power, there were still some intrinsic limitations. For example, the sample size of this study is relatively small. Subsequently, we will pay more attention to the influence of *IFNG* polymorphisms on PTB risk.

In summary, our study revealed *IFNGR1* gene may have a significant association with the PTB risk in the Chinese Tibetan population, and provides theoretical foundation for personalized medicine of Tibetan PTB patients. Our findings need further verified with studies with enlarge sample size, and future studies are required to determine the functional consequences of these polymorphisms in pulmonary tuberculosis and the biological mechanism underlying this association.

## MATERIALS AND METHODS

### Study participants

This case-control study consisted of 467 cases of tuberculosis and 503 healthy controls. Tibetan PTB patients were recruited from the third Hospital of Tibet Autonomous Region from October 2012 to September 2013 in Lhasa, China. The diagnosis of PTB was based on clinical radiological, sputum acid-fast bacillus smear positivity, culture, and response to antituberculosis chemotherapy as described previously [23, 24]. At the same time, stochastic samples of 503 healthy controls were enlisted from the same geographical origin and were living in the same region as the patients with PTB. The controls had no previous clinical history or laboratory criteria suggestive of PTB infection. All subjects were all Tibetan Chinese living in Lhasa and nearby.

Subjects who possessed chronic inflammatory and conditions involving vital organs such as the HIV positive and known to present any autoimmune, family history of cancer or other diseases were excluded from this study. For controls, a standardized epidemiological questionnaire including residential region, age, smoking status, alcohol use, ethnicity and education level was used to collect personal data. As for patients, we collected related information through consultation with treating physicians or medical chart review. Unfortunately, in addition to age and gender, a considerable number of participates had no relevant information containing smoking status and alcohol use.

### Ethics committee statement

This case-control study was performed in compliance with the principles of the Declaration of Helsinki of the World Medical Association and obtained the permission from the Ethics Committee of the third Hospital of Tibet Autonomous Region. All of the participants were informed of the case-control study and their consents were obtained.

### SNPs selection and genotyping

According to the past studies [4, 11, 17], rs9376267, rs9376268, rs1327475, rs7749390 and rs1327474 had been found study with the risk of PTB in other populations. Additionally, rs2069727, rs1861493, rs1861494 and rs2069705 in *IFNG* were also investigated the connection with the PTB risk in this present study.

We collected blood sample from each patient during laboratory examination after their recruitment. DNA was extracted from whole blood samples using the Gold Mag-Mini Whole Blood Genomic DNA Purification Kit [25]. DNA concentration was measured by spectrometry (DU530 UV/VIS spectrophotometer, Beckman Instruments, Fullerton, CA, USA). We used Sequenom MassARRAY Assay Design 4.0 Software (Agena Bioscience Inc.) to design a Multiplexed SNP Mass-EXTEND assay [26]. The SNPs were genotyped with a Sequenom MassARRAY RS1000 using the standard protocol recommended by the manufacturer [26]. Data management and analysis were performed using Sequenom Typer 4.0 software (Agena Bioscience Inc) [26, 27].

### Statistical analysis

We used the SPSS 21.0 statistical packages (SPSS Inc., Chi-cago, IL, USA) and Microsoft Excel for statistical analysis. All *P*-values in this study were two sided, and *P*<0.05 was considered the threshold for statistical significance. We tested for differences in tSNP genotype distribution between cases and controls using the χ^2^ test [28]. Each SNP of the genotype frequencies in control subjects were checked by using Hardy-Weinberg equilibrium (HWE). Allele and genotype frequencies for each SNP of PTB patients and control subjects were compared using Chi-square test. Odds ratios (ORs) and 95% confidence intervals (CIs) were tested by unconditional logistic regression analysis to evaluate the effects of the polymorphisms on the risk of PTB [29]. Therefore, Bonferroni's multiple adjustment was applied to the level of significance, which was set at P < 0.0011 (0.05/45). Finally, we used SNP stats, website software to test the associations between certain SNPs and the risk of PTB in three models (dominant, recessive, and additive). The Haploview software package (version 4.2) and SHEsis software platform (http://analysis.bio-x.cn) were used to assess linkage disequilibrium, haplotype construction, and the genetic association between polymorphisms, with a D’ > 0.8 indicating that related SNPs formed a single block [30].

## Abbreviations

PTB: Pulmonary Tuberculosis
SNP: Single nucleotide polymorphism
ORs: Odds ratios
CI: Confidence intervals
LD: Linkage disequilibrium
